# Prevalence and risk of cognitive impairment among patients with asthma: A systematic review and meta‐analysis

**DOI:** 10.1002/brb3.70048

**Published:** 2025-02-27

**Authors:** Ganesh Bushi, Mahalaqua Nazli Khatib, Shilpa Gaidhane, Renuka Jyothi. S, Manish Srivastava, Apurva Koul, M. Ravi Kumar, Quazi Syed Zahiruddin, Sarvesh Rustagi, Sanjit Sah, Hashem Abu Serhan, Muhammed Shabil

**Affiliations:** ^1^ Center for Global Health Research, Saveetha Medical College and Hospital, Saveetha Institute of Medical and Technical Sciences Saveetha University Chennai India; ^2^ Evidence for Policy and Learning Global Center for Evidence Synthesis Chandigarh India; ^3^ Division of Evidence Synthesis, Global Consortium of Public Health and Research Datta Meghe Institute of Higher Education Wardha India; ^4^ One Health Centre (COHERD), Jawaharlal Nehru Medical College Datta Meghe Institute of Higher Education Wardha India; ^5^ Department of Biotechnology and Genetics, School of Sciences JAIN (Deemed to be University) Bangalore Karnataka India; ^6^ Department of Endocrinology NIMS University Jaipur India; ^7^ Chandigarh Pharmacy College Chandigarh Group of College Mohali Punjab India; ^8^ Department of Chemistry Raghu Engineering College Visakhapatnam Andhra Pradesh India; ^9^ South Asia Infant Feeding Research Network (SAIFRN), Division of Evidence Synthesis, Global Consortium of Public Health and Research Datta Meghe Institute of Higher Education Wardha India; ^10^ School of Applied and Life Sciences Uttaranchal University Dehradun Uttarakhand India; ^11^ Department of Paediatrics, Dr. D. Y. Patil Medical College, Hospital and Research Centre Dr. D. Y. Patil Vidyapeeth Pune Maharashtra India; ^12^ Department of Public Health Dentistry, Dr. D.Y. Patil Dental College and Hospital Dr. D.Y. Patil Vidyapeeth Pune Maharashtra India; ^13^ Department of Ophthalmology Hamad Medical Corporation Doha Qatar; ^14^ University Center for Research and Development Chandigarh University Mohali Punjab India; ^15^ Medical Laboratories Techniques Department AL‐Mustaqbal University Hillah Babil Iraq

**Keywords:** Alzheimer's disease, asthma, cognitive decline, dementia, meta‐analysis, systematic review

## Abstract

**Background:**

Asthma, a prevalent chronic respiratory condition, is hypothesized to influence cognitive health; however, the precise nature of this association remains unclear. This systematic review and meta‐analysis aimed to elucidate the prevalence and risk of cognitive impairment in individuals with asthma.

**Methods:**

A comprehensive literature search was performed in databases such as PubMed, EMBASE, and Web of Science, spanning publications up to December 25, 2023. This search aimed to identify studies that assessed cognitive impairment in patients with asthma. We used the random effects model in the R v4.3 software for the meta‐analysis to evaluate the prevalence and risk of cognitive decline, including dementia and Alzheimer's disease, among asthma patients. To ensure robustness and validity, the quality of the studies was assessed using Newcastle–Ottawa scale.

**Results:**

Twelve studies met the inclusion criteria, of these 10 were eligible for meta‐analysis. The pooled prevalence of cognitive impairment in patients with asthma was 16.3%. The analysis also revealed an increased hazard ratio of 1.47 (95% confidence interval [1.09; 1.84]) for cognitive impairment in patients with asthma compared to the control group (individuals without asthma). Significant heterogeneity and publication bias were observed across the studies. The results underscored the substantial correlation between asthma and heightened risks of cognitive decline, dementia, and Alzheimer's disease.

**Conclusion:**

This review found a notable association between asthma and an increased risk of cognitive decline, including dementia and Alzheimer's disease. These findings highlight the importance of integrating cognitive health assessments into asthma care. Further research is required to understand this relationship and develop effective treatments. Emphasizing a holistic approach to asthma management, these findings highlight the need to consider both respiratory and cognitive health for comprehensive patient care.

## Graphical abstract

This systematic review and meta‐analysis assessed the prevalence and risk of cognitive impairment in asthma patients, finding a 16.3% prevalence and a 1.47‐fold increased risk compared with controls. Asthma is linked to dementia and Alzheimer's disease, underscoring the need for cognitive assessments in asthma care. Further research is necessary.

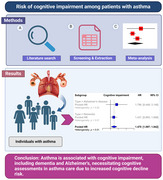

## 1 INTRODUCTION

Asthma, a chronic inflammatory respiratory disease, significantly impacts global health, affecting an estimated 339 million people worldwide (Asher et al., 2020). This condition, characterized by symptoms such as wheezing, breathlessness, and coughing, primarily during night or early morning, extends beyond respiratory impairment, potentially influencing cognitive health (Irani et al., 2017). With its increasing prevalence, particularly in developed nations, and considerable healthcare burden, understanding the broader impact of asthmas, including its effects on cognitive function, has become imperative. Cognitive functions, encompassing learning, thinking, reasoning, and memory, are crucial for daily life (Ren et al., 2021). Investigating the link between asthma and cognitive function is critical amid rising global concerns regarding cognitive decline and dementia. Globally, cognitive decline and dementia are significant public health challenges (Nair et al., 2022). With around 50 million people affected by dementia as of 2020, and projections suggesting a potential tripling of this number by 2050, the impact of cognitive disorders is profound (Lovett et al., 2020; MacLeod et al., 2021). These conditions not only affect individuals but also have substantial implications for families, healthcare systems, and economies, with the global cost of dementia exceeding $1 trillion annually (Lovett et al., 2020).

Several studies have reported that the relationship between asthma and cognitive decline is complex and potentially rooted in the systemic inflammatory characteristics of asthma. This chronic airway inflammation might not only affect respiratory health but could also influence brain function and cognition (Goel et al., 2024; Nair et al., 2023). Central to this are mechanisms such as chronic intermittent hypoxia during asthma exacerbations, which may damage critical brain areas such as the hippocampus, impairing cognitive functions (Kroll et al., 2018). Systemic inflammation in asthma also contributes to cognitive deficits, with inflammatory cytokines potentially disrupting brain function. Moreover, molecular changes, including alterations in HIF‐1α and HIF‐2α levels, involved in oxygen regulation, are also believed to play a role in cognitive impairments in asthma patients (Bushi et al., 2023; Y. Wang, Mou, et al., 2023). A comprehensive understanding of these mechanisms is vital for developing effective interventions to manage respiratory symptoms and cognitive health inpatients with asthmas.

Understanding the prevalence and risk of cognitive impairment in individuals with asthma is crucial for several reasons. First, it can help to identify vulnerable populations that may require targeted interventions to mitigate the risk of cognitive decline. Second, it can inform healthcare providers about the potential long‐term consequences of asthma beyond its respiratory manifestations, thus emphasizing the need for comprehensive patient care. Third, this study can contribute to the development of preventive strategies and therapeutic approaches aimed at preserving cognitive function in patients with asthma. Therefore, the potential association between asthma, a common chronic condition, and cognitive decline requires urgent attention. This systematic review and meta‐analysis sought to synthesize existing research to understand the prevalence and risk of cognitive decline among asthma patients, aiming to provide insights crucial for patient care and public health strategy development.

## 2 METHODS

We followed the Preferred Reporting Items for Systematic Reviews and Meta‐Analyses (PRISMA) guidelines in this systematic review and meta‐analysis (Shamim et al., 2023) to investigate the prevalence and association between asthma and cognitive impairments, including dementia, Alzheimer's disease, memory loss, and confusion (Table S1). We conducted this review using the Nested Knowledge software and registered our study protocol with PROSPERO.

### 2.1 Selection criteria

The studies examining cognitive impairment in patients diagnosed with asthma were included without restrictions on age, sex, geographical location, or language. The primary focus was on determining the prevalence of cognitive impairment and its association with asthma. We considered various types of studies, including randomized controlled trials, cohort studies, case–control studies, and observational studies, published up to December 25, 2023. Detailed inclusion criteria are outlined in Table S2.

### 2.2 Database search

A thorough literature search was conducted in PubMed, EMBASE, and Web of Science, covering publications from the beginning up to December 25, 2023. Our search strategy involved a mix of keywords and medical subject headings, focusing on “asthma,” “cognitive impairment,” “dementia,” “Alzheimer's,” “memory loss,” and “confusion.” To ensure comprehensive coverage of the relevant literature, no filters or restrictions were applied during the search. Additionally, we conducted a citation search to identify further articles. A detailed description of the search strategy is provided in Table S3.

### 2.3 Screening

This review focuses on asthma and its cognitive implications, and the screening of articles was meticulously performed by two independent evaluators using the Nested Knowledge software (Nested Knowledge). This initial phase involved scrutinizing the titles and abstracts to identify studies that were not pertinent to our research objectives. Following this, a more detailed examination of the full texts was undertaken for studies that appeared potentially relevant. In instances of discordance between the two reviewers, a third independent reviewer mediated and resolved these differences, thereby ensuring the integrity and objectivity of the review process. This rigorous method of article selection is essential for upholding the scholarly standards of systematic reviews and meta‐analyses.

### 2.4 Data extraction

Two reviewers independently performed the data extraction using a standardized MS Excel spreadsheet. The collected data encompassed a variety of study characteristics, such as study design, country of origin, demographic information of the participants, types of cognitive impairments studied, total number of participants, and prevalence rates of these cognitive impairments. This process included collating comprehensive details about the total number of participants and those who exhibited cognitive impairment following an asthma diagnosis. We utilized the Nested Knowledge software's “tagging” feature for efficient data extraction and systematically organized the data in Microsoft Excel. Discrepancies in data extraction were resolved through discussions among the research team or consultation with a third reviewer.

### 2.5 Quality assessment

The methodological quality and risk of bias of the observational studies were appraised using the Newcastle–Ottawa scale (NOS). This scale, specifically designed to evaluate non‐randomized studies, assesses studies based on their selection of groups, comparability of groups, and ascertainment of outcomes. It considers critical factors, such as the representativeness of the cohorts, accuracy of exposure measurements, control of confounding factors, and reliability of outcome reporting. The NOS provides a score of nine points, which serves as an indicator of the overall quality of each study included in our review.

### 2.6 Data analysis

All statistical analyses were performed using R software version 4.3 (Shamim et al., 2023). We employed a random‐effects model in our meta‐analysis to integrate the data on both the prevalence and association of cognitive impairment in the asthma groups. This approach allowed us to assess the risk ratio of cognitive impairment while accommodating for study variances. Heterogeneity among studies was quantitatively assessed using *I* ^2^ and Tau^2^ statistics, with Tau^2^ determined using the maximum likelihood method (Gandhi et al., 2023; Langan et al., 2019). We included 95% prediction intervals in our analysis and set a statistical significance threshold at *p* < .05. Additionally, we conducted a publication bias assessment using funnel plot analysis and performed a sensitivity analysis using a leave‐one‐out method to evaluate the influence of individual studies on the overall results.

## 3 RESULTS

### 3.1 Literature search and study selection

**FIGURE 1:**
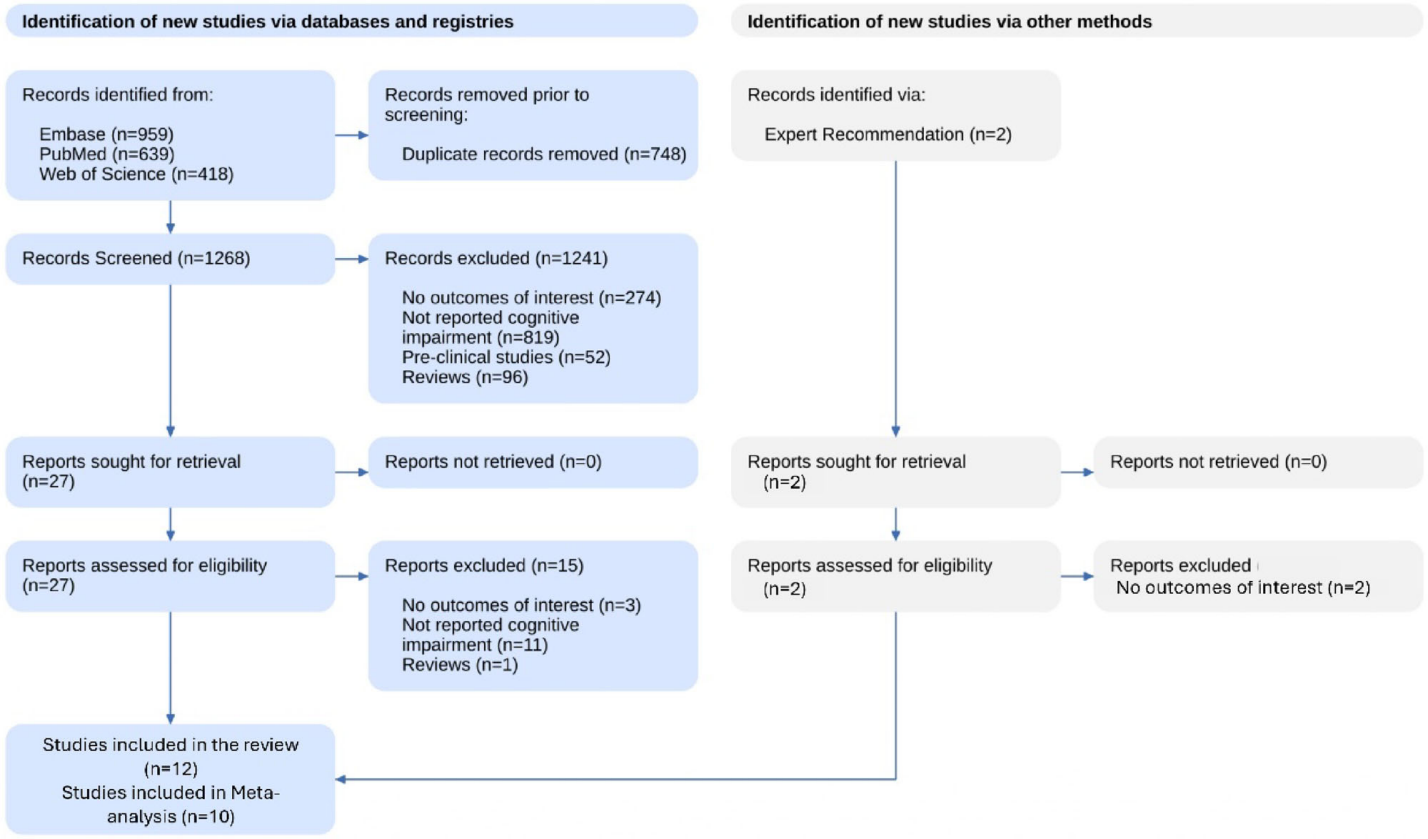
Flow diagram representing the screening and selection process of the studies.

Figure 1 PRISMA flow diagram provides a detailed overview of the study selection process for systematic review and meta‐analysis. Initially, 2016 records were identified through database search. To manage duplicate entries, 748 records were removed, leaving 1268 records to be screened. Of these, 1241 records were excluded after screening titles and abstracts for reasons such as irrelevance to the outcomes of interest, preclinical studies, reviews, or other non‐relevant work, resulting in 27 records. These 27 articles underwent full‐text retrieval for closer examination, and 15 were further excluded because they did not report the outcomes of interest or were review articles. Furthermore, two studies were discovered using methods such as citation search, but these were not included because they failed to meet our inclusion criteria. Ultimately, 12 studies were included in the qualitative analysis; of these, 10 were eligible for inclusion in the meta‐analysis.

### 3.2 Characteristics of Included Studies

Table 1 presents the summary characteristics of the studies included in the review, which encompass a range of studies from diverse geographical locations such as Korea, Taiwan, the USA, Finland, Saudi Arabia, and Poland. This global perspective highlights the relationship between asthma and cognitive impairment. The research methodologies varied, including prospective and retrospective observational studies, population‐based studies, cross‐sectional studies, and case–control studies. The studies explored a broad age range of participants, with some focusing on older adults and others including a wider age spectrum. The cognitive impairments investigated included all‐cause dementia, Alzheimer's disease, vascular dementia, general cognitive impairment, mild cognitive impairment, and dementia. Sample sizes ranged from as few as 18 participants to over six million, providing comprehensive insights into the relationship between asthma and cognitive decline and dementia. Furthermore, the quality assessment using the NOS indicated that the overall quality of the included studies was moderate to high.

**TABLE 1:** Characteristics of included studies.

Study	Country	Study design	Age (mean)	Male (%)	Type of cognitive impairment	Total sample	Key findings
Abuaish et al. (2023)	Saudi Arabia	Cross‐sectional study	52	22.60	Mild cognitive impairment	243	Asthma management impacts cognitive health in middle‐aged and elderly individuals in Saudi Arabia.
Bernhoff et al. (2022)	Denmark	Cohort study	NA	NA	Cognitive impairment	18	Severe eosinophilic asthma is linked to impairments in verbal learning and memory.
Bozek et al. (2010)	Poland	Cross‐sectional study	64	38.9	Dementia, Mild cognitive impairment	359	Asthma management may improve cognitive functions in individuals with cognitive impairments.
Bratek et al. (2015)	Poland	Cross‐sectional study	51	NA	Dementia	59	Asthma and COPD patients have higher rates of depressive, anxiety symptoms, and cognitive dysfunctions compared to controls.
Caldera‐Alvarado et al. (2013)	USA	Cross‐sectional study	≥55	72.50	Mild cognitive impairment	1380	Significant correlation between asthma and cognitive impairment in older adults (OR = 1.78).
Chen et al. (2014)	Taiwan	Case–control study	60.88	41.7	Alzheimer's disease, Unspecified dementia	55,150	Asthma increases the risk of dementia, including Alzheimer's disease.
Joh et al. (2022)	Korea	Prospective observational study	54.4	49.90	All cause dementia, Alzheimer's disease, Vascular dementia	6,785,948	Asthma and allergic conditions are linked to a higher risk of all‐cause dementia and its subtypes, with a dose‐response relationship.
Kim et al. (2019)	Korea	Case–control study	≥60‐year‐old	32	Dementia	11,442	No significant association between asthma and neurodegenerative dementia (OR = 0.97).
Nair et al. (2022)	USA	Retrospective observational	63.7	NA	Dementia	375	Severe asthma may increase dementia risk, especially with cardiovascular disease or genetic susceptibility.
Peng et al. (2014)	Taiwan	Retrospective observational study	53.8	45.7	Dementia	63,855	Asthma is associated with a higher risk of dementia, escalating with more frequent asthma exacerbations.
Rhyou and Nam (2021)	Korea	Retrospective observational study	>18 years	NA	Cognitive impairment	202	Asthma is linked to cognitive impairment, especially in older adults and those with a longer disease duration.
Rusanen et al. (2013)	Finland	Population based study	NA	NA	Dementia	2000	Midlife asthma and COPD nearly double the risk of mild cognitive impairment (MCI) and dementia later in life.

Abbreviation: COPD, chronic obstructive pulmonary disease.

### 3.3 Meta‐analysis

#### 3.3.1 Prevalence of cognitive impairment in asthma patients

In the meta‐analysis involving a total of 500,502 participants, 43,818 reported events of cognitive impairments (composite), resulting in a pooled prevalence of 16.3% with a 95% confidence interval (CI) of [0.08; 0.29.] from eight studies. The prediction interval was quite broad, ranging from 1.6% to 69.5%, indicating substantial variability that could be expected in future studies. Overall heterogeneity was high (*I* ^2^ = 99%), suggesting significant differences across the studies included.

**FIGURE 2:**
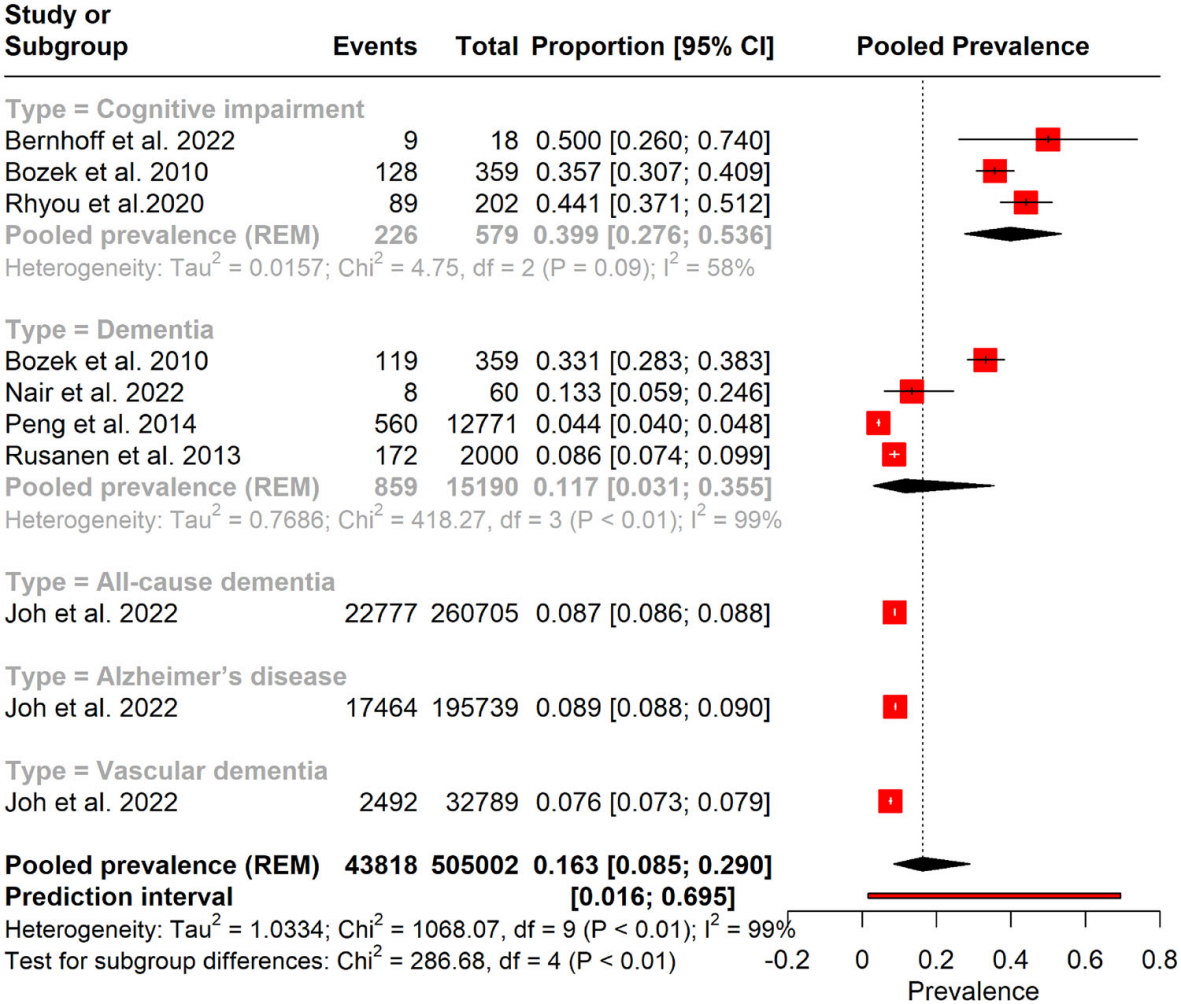
Forest plot depicting the pooled prevalence of cognitive impairment in asthma. CI, confidence interval.

The subgroup analysis of the prevalence of cognitive impairment was 39.9% (95% CI [0.276; 0.536]), dementia was 11.7% (95% CI [0.031; 0.355]), all‐cause dementia was 8.7% (95% CI [0.086; 0.088]), vascular dementia was 7.6% (95% CI [0.073; 0.079]), and Alzheimer's disease was 8.9% (95% CI [0.088; 0.090]). The confidence intervals for each subgroup highlight the differences in prevalence rates, emphasizing the variation in the impact of asthma on different forms of cognitive disorders (*p*‐value < .01) (Figure 2).

#### 3.3.2 The association between cognitive impairment in asthma patients

The pooled analysis of studies investigating the risk of cognitive decline in patients with asthma revealed an overall hazard ratio (HR) of 1.470 (95% CI of [1.097−1.842]). This indicates a 47% increased risk of cognitive impairment in individuals with asthma compared with those without asthma. The analysis was characterized by substantial heterogeneity (*I* ^2^ = 91%) and a prediction interval of [0.236; 2.704], suggesting that the actual risk of cognitive decline associated with asthma could vary significantly between studies.

**FIGURE 3:**
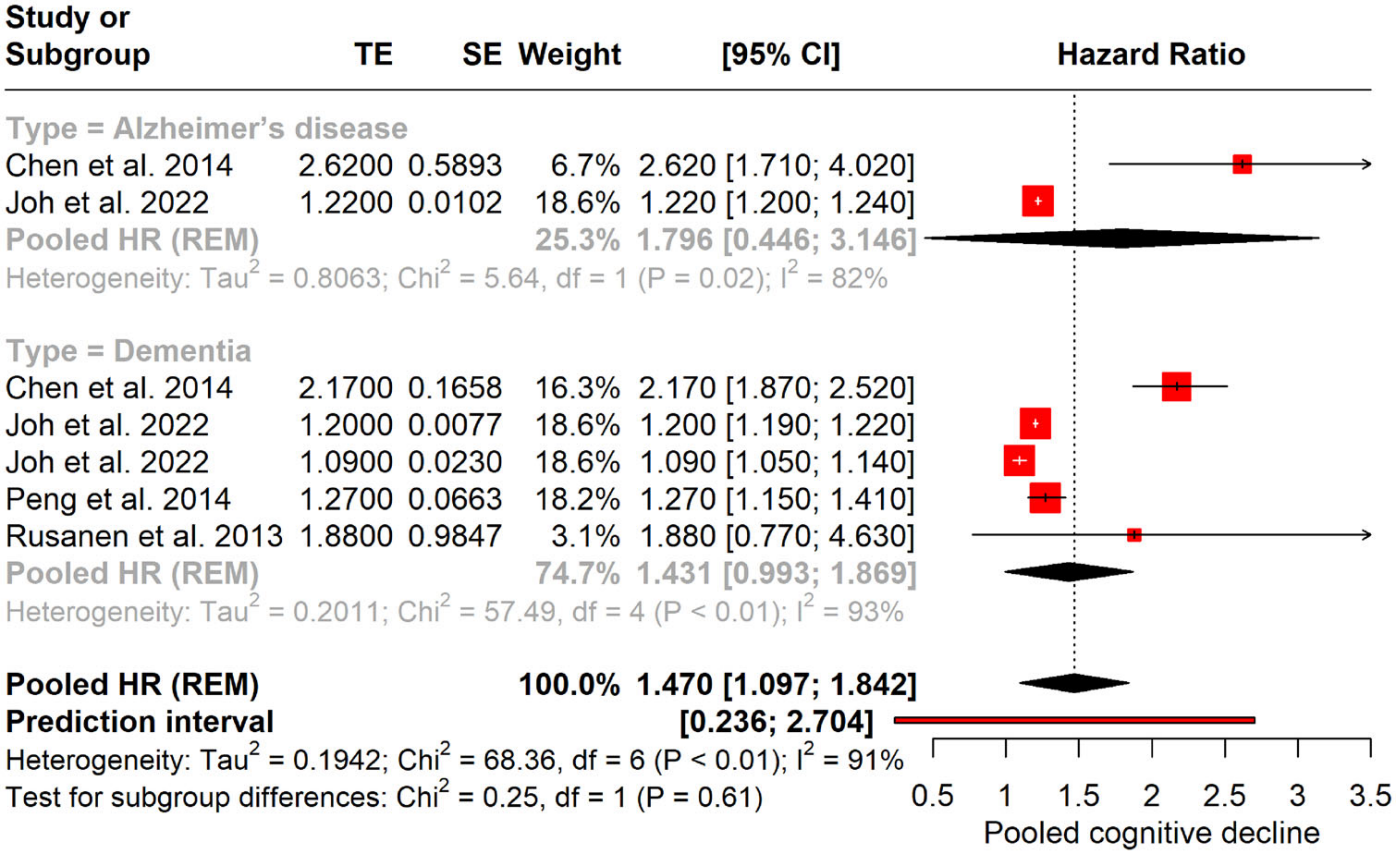
Forest plot showing the risk of cognitive impairment in patients with asthma. HR, hazard ratio; REM, random‐effects model; LFK, luis furuya‐kanamori.

When examining the subgroups, the pooled HR for Alzheimer's disease was 2.620 (95% CI [1.710; 4.020]), indicating more than a doubling of risk, whereas for dementia, the pooled HR was 1.431 (95% CI [0.993; 1.869]), showing a 43% increased risk. These subgroup results highlight a notable difference in the strength of the association between asthma and the different types of cognitive impairment. The heterogeneity within these subgroups remains high (*I* ^2^ = 82% for Alzheimer's disease and *I* ^2^ = 91% for dementia), reflecting variations in study designs or populations. The observed heterogeneity underscores the need for cautious interpretation of the increased risk and consideration of the individual study characteristics (Figure 3).

#### 3.3.3 Sensitivity analysis and publication bias

**FIGURE 4:**
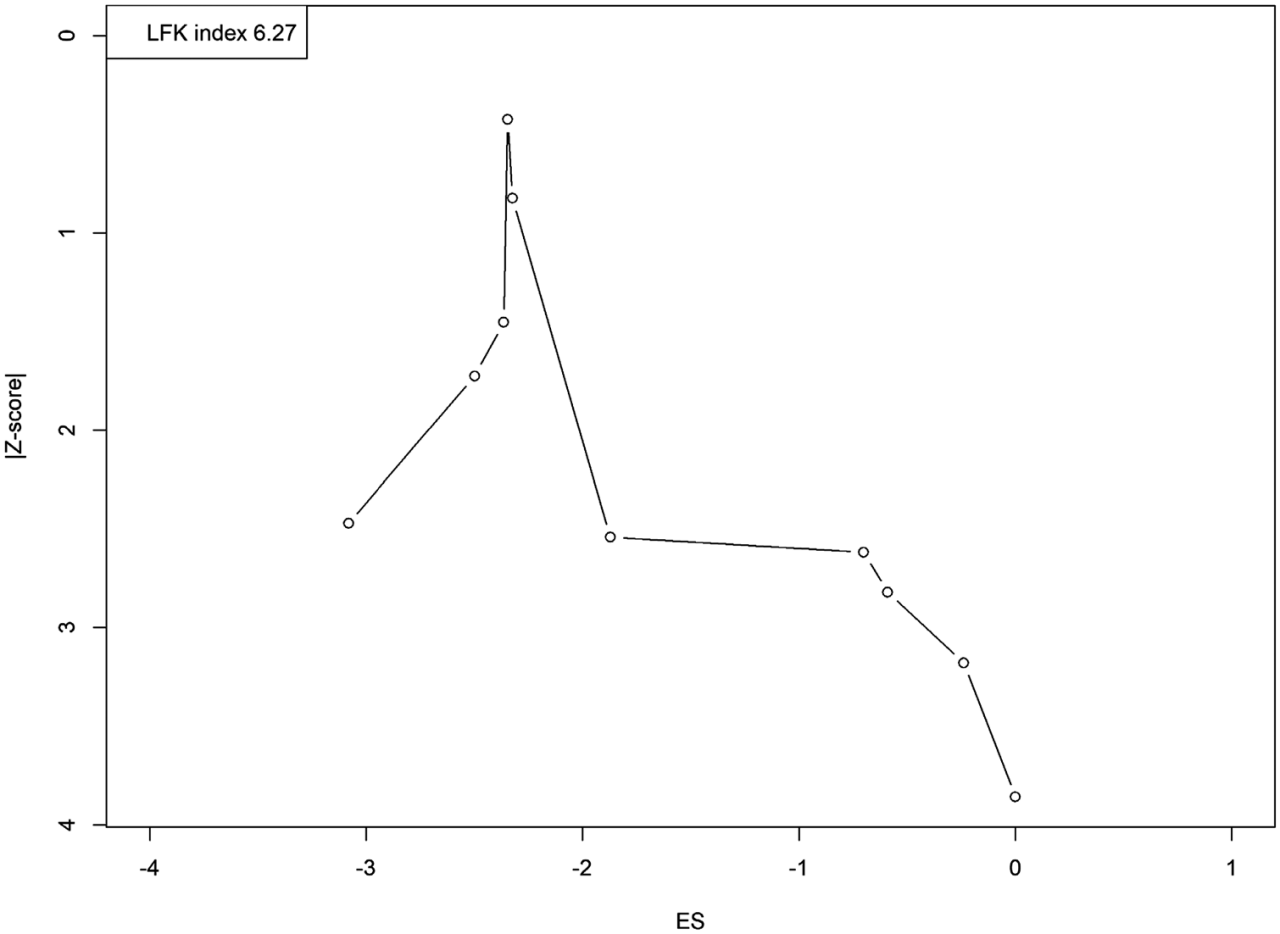
Doi plot depicting the publication of included studies.

The sensitivity analysis depicted in the forest plot indicates that the pooled prevalence of cognitive impairment among patients with asthma is relatively stable across the “leave‐one‐out” approach. Variations in the prevalence estimates remain modest when individual studies are omitted, with Peng et al. (2014) showing the largest influence on increasing prevalence and Rhyou and Nam (2021) the least. Overall, the consistency of the pooled prevalence of 16.3% suggests that the meta‐analysis results are robust and not overly dependent on any single study (Figure S1). The Doi and funnel plots suggest potential publication bias in the meta‐analysis, as indicated by the asymmetrical distribution of studies and an luis furuya‐kanamori (LFK) index of 6.27, which is considerably higher. This skewness implies that smaller studies with less significant results might be underrepresented in the analysis, potentially overestimating the effect size due to the preferential publication of studies with positive outcomes (Figure 4 and Figure S2).

## 4 DISCUSSION

The systematic review and meta‐analysis conducted here presented a significant correlation between asthma and an increased risk of cognitive decline, with a pooled hazard ratio suggesting a 47% elevated risk for cognitive impairment among asthma patients compared to the control group, and a high pooled prevalence of 16.4% was found across studies. This association is particularly pronounced in Alzheimer's disease, where the risk is extremely high. This association is largely driven by the chronic inflammatory characteristic of asthma, which can extend beyond the airways to affect the brain, potentially leading to neuroinflammation. Severe asthma exacerbates this link by causing intermittent hypoxia, damaging brain tissues that are highly sensitive to oxygen deprivation and impacting critical areas like the hippocampus involved in memory and learning (T. Wang, Huang, et al., 2023). Additionally, asthma treatments, particularly corticosteroids, can affect cognitive functions by disrupting neurotransmitter levels and affecting the hypothalamic–pituitary–adrenal axis. Moreover, age‐related changes in lung function and brain physiology complicate the relationship between asthma and cognitive decline, with older adults being more susceptible. Comorbid conditions common in asthma patients, such as cardiovascular disease, diabetes, and obesity, further add to the risk of cognitive decline (Y. Wang, Mou, et al., 2023).

Several studies, including those by Peng et al. (2014) and Caldera‐Alvarado et al. (2013), have provided substantial insights into the relationship between asthma and cognitive decline. Peng et al. found that asthma patients have a 1.27‐fold higher risk of developing dementia than the general population after adjusting for various confounders. This finding suggests a potential connection between chronic inflammatory conditions such as asthma and cognitive decline. Similarly, Caldera‐Alvarado et al. (2013) reported a significant 78% increase in the risk of mild cognitive impairment in older individuals with asthma, further underscoring this link. Research conducted by Rhyou and Nam (2021) has delved deeper into the complexities of this interplay between asthma and cognitive function. Their findings indicated an increased prevalence of cognitive dysfunction in patients with asthma across different age groups. This study highlights the susceptibility of brain tissue to fluctuations in oxygen levels, suggesting that asthma attacks and subsequent cerebral hypoxia could lead to neural abnormalities and cognitive deficits. Crucially, Rhyou and Nam also highlighted a possible bidirectional relationship between asthma and cognitive function. They suggested that effective control of asthma could lead to improved cognitive outcomes, whereas cognitive deficits may adversely affect asthma management. This nuanced understanding of the relationship is vital for developing comprehensive care strategies for patients with asthma, particularly for addressing cognitive health concerns (Rhyou & Nam, 2021).

Surprisingly, a study conducted by Kim et al. (2019) employed a nested case–control design in an older adult population and found no significant increase in the risk of neurodegenerative dementia linked to asthma. Although Montreal Cognitive Assessment (MoCA) test scores were lower in asthmatics (57%) compared to non‐asthmatics (42%), the scores did not reach statistical significance. However, after adjusting for age, body mass index (BMI), and gender, they found a significant association between asthma and mild cognitive impairment (MCI), with an odds ratio of 1.80. This significant association persisted even after adjusting for chronic health conditions such as heart disease, hypertension, and diabetes. These variations in findings underscore the need for further robust research to clarify the nature of the relationship between asthma and dementia and to identify the underlying mechanisms contributing to this association in different patient populations (Kim et al., 2019).

Interestingly, a study conducted by Kim et al. employed a nested case–control design in an older adult population and found no significant increase in the risk of neurodegenerative dementia linked to asthma. MoCA test scores were lower in asthmatics compared to non‐asthmatics, and the scores did not reach statistical significance. MCI was prevalent in 47% of the study population, with a higher prevalence in asthmatics (57%) compared to non‐asthmatics (42%). However, after adjusting age, BMI, and gender, they found a significant association between asthma and MCI after controlling for, with an odds ratio of 1.80. This significant association persisted even after adjusting for chronic health conditions such as heart disease, hypertension, and diabetes. This variation in findings underscores the need for further robust research to clarify the nature of the relationship between asthma and dementia and to identify the underlying mechanisms contributing to this association in different patient populations.

Despite the robust findings, several limitations of the evidence included in this review should be acknowledged. Substantial heterogeneity across the studies, as indicated by high *I* ^2^ values, suggests variability in outcomes due to differences in study populations, diagnostic criteria for cognitive impairment, and asthma treatment regimens. Additionally, the potential for publication bias, as indicated by asymmetrical funnel plots and a high LFK index, might result in an overrepresentation of studies with positive findings, thus overestimating the effect size. Furthermore, the predominantly observational nature of the included studies limits the ability to establish causality between asthma and cognitive impairment, underscoring the need for more randomized controlled trials in this area.

The results of this review have significant implications for clinical practice, policy development, and future research. In clinical practice, the findings highlight the need to integrate cognitive health assessments into routine asthma care. This holistic approach ensures that healthcare providers manage both respiratory and cognitive health, potentially leading to early detection and intervention for cognitive decline, dementia, and Alzheimer's disease in asthma patients. Regular cognitive assessments using tools such as the MoCA could become standard in asthma management protocols (Amaljith et al., 2024; Iamthanaporn et al., 2023). Additionally, creating tailored treatment plans that address both respiratory and cognitive health could significantly enhance patient outcomes and quality of life.

From a policy perspective, these findings suggest updating healthcare guidelines to include cognitive health evaluations for individuals with asthma. Policymakers should promote training programs for healthcare providers on the cognitive risks associated with asthma and the importance of comprehensive care strategies. Insurance providers and healthcare systems may need to adjust their coverage to include cognitive assessments and interdisciplinary care approaches. Public health campaigns could also raise awareness about the link between asthma and cognitive impairment, encouraging individuals with asthma to seek comprehensive care. Future research should focus on understanding the mechanisms behind the asthma‐cognitive decline link through longitudinal studies and randomized controlled trials. Investigating the role of systemic inflammation, hypoxia, and medication effects on brain health, as well as the efficacy of integrated care models, will be crucial. Additionally, examining the variability in cognitive impairment risk among different asthma populations will help develop personalized treatment approaches.

## 5 CONCLUSION

A significant association between asthma and a higher risk of cognitive decline, including dementia and Alzheimer's disease, highlights the crucial need to include cognitive health assessments in asthma care. Further research is needed to understand the underlying factors of this association and develop effective treatments. These findings emphasize the importance of a comprehensive approach to managing asthma that addresses both respiratory and cognitive well‐being. Such an approach ensures thorough patient care, acknowledging the interplay between asthma and cognitive impairment, and strives to improve overall health outcomes.

## AUTHOR CONTRIBUTIONS

**Ganesh Bushi**: Conceptualization; methodology; software; data curation; writing—review and editing. **Mahalaqua Nazli Khatib**: Conceptualization; methodology; software; data curation; project administration. **Shilpa Gaidhane**: Conceptualization; investigation; visualization; writing—review and editing. **Renuka Jyothi. S**: Conceptualization; investigation; formal analysis; visualization; writing—review and editing. **Manish Srivastava**: Conceptualization; visualization; writing—review and editing; software; supervision. **Apurva Koul**: Conceptualization; writing—review and editing; validation; software. **M Ravi Kumar**: Conceptualization; validation; project administration; resources; software. **Quazi Syed Zahiruddin**: Software; methodology; conceptualization; writing—original draft; writing—review and editing; project administration. **Sarvesh Rustagi**: Conceptualization; data curation; resources; validation; writing—review and editing; writing—original draft. **Sanjit Sah**: Conceptualization; writing—original draft; writing—review and editing. **Hashem Abu Serhan**: Writing—original draft; writing—review and editing; conceptualization; funding acquisition; project administration. **Muhammed Shabil**: Conceptualization; visualization; writing—review and editing; project administration.

## CONFLICT OF INTEREST STATEMENT

The authors declare no conflicts of interest.

### PEER REVIEW

The peer review history for this article is available at https://publons.com/publon/10.1002/brb3.70048.

## FUNDING INFORMATION

This study received no external funding.

## Supporting information




**Table S1**. PRISMA Checklist.
**Table S2**. Inclusion and Exclusion criteria.
**Table S3**. The adjusted search terms as per searched electronic databases.
**Table S4**. Newcastle‐Ottawa Scale for the quality assessment of included studies.
**Figure S1**. Leave one out analysis of the studies.
**Figure S2**. The funnel plot represents the publication bias of the studies.

## Data Availability

All data used in this review are provided in the manuscript and supplementary files.

## References

[brb370048-bib-0001] Abuaish, S. , Eltayeb, H. , Bepari, A. , Hussain, S. A. , Alqahtani, R. S. , Alshahrani, W. S. , Alqahtani, A. H. , Almegbil, N. S. , & Alzahrani, W. N. (2023). The association of asthma with anxiety, depression, and mild cognitive impairment among middle‐aged and elderly individuals in Saudi Arabia. Behavioral Sciences, 13(10), 842. 37887495 10.3390/bs13100842 PMC10604786

[brb370048-bib-0002] Amaljith, A. B. , Marzo, R. R. , Lekamwasam, S. , Kisa, A. , Behera, A. , Preetha, S. , Saravanan, P. B. , Shah, P.B. , Mahapatra, S. S. , Kumbha, G. , Vishaal, P. , & Rajagopal, V. (2024). Prevalence of fall and its associated factors among elderly population in India: Evidence from the Longitudinal Aging Study of India (LASI). The Evidence, 2(2).

[brb370048-bib-0003] Asher, M. I. , García‐Marcos, L. , Pearce, N. E. , & Strachan, D. P. (2020). Trends in worldwide asthma prevalence. European Respiratory Journal, 56(6), 2002094. 32972987 10.1183/13993003.02094-2020

[brb370048-bib-0004] Bernhoff, C. , Jespersen, A. , Dyhre‐Petersen, N. , Klein, D. , Miskowiak, K. , & Porsbjerg, C. (2022). Cognitive impairment is common in patients with severe astma that are commenced on a biological treatment. European Respiratory Society.

[brb370048-bib-0005] Bozek, A. , Krajewska, J. , & Jarzab, J. (2010). The improvement of cognitive functions in patients with bronchial asthma after therapy. Journal of Asthma, 47(10), 1148–1152. 10.3109/02770903.2010.513077 21039205

[brb370048-bib-0006] Bratek, A. , Zawada, K. , Beil‐Gawełczyk, J. , Beil, S. , Sozańska, E. , Krysta, K. , Barczyk, A. , Krupka‐Matuszczyk, I. , & Pierzchała, W. (2015). Depressiveness, symptoms of anxiety and cognitive dysfunctions in patients with asthma and chronic obstructive pulmonary disease (COPD): Possible associations with inflammation markers: A pilot study. Journal of Neural Transmission, 122, 83–91. 10.1007/s00702-014-1171-9 PMC4529448 24532256

[brb370048-bib-0007] Bushi, G. , Padhi, B. K. , Shabil, M. , Satapathy, P. , Rustagi, S. , Pradhan, K. B. , Al‐Qaim, Z. H. , Khubchandani, J. , Sah, R. , Sah, S. , & Anand, A. (2023). Cardiovascular disease outcomes associated with obstructive sleep apnea in diabetics: A systematic review and meta‐analysis. Diseases, 11(3), 103. 37606474 10.3390/diseases11030103 PMC10443251

[brb370048-bib-0008] Caldera‐Alvarado, G. , Khan, D. , Defina, L. , Pieper, A. , & Brown, E. (2013). Relationship between asthma and cognition: The Cooper Center Longitudinal Study. Allergy, 68(4), 545–548. 23409872 10.1111/all.12125

[brb370048-bib-0009] Chen, M.‐H. , Li, C.‐T. , Tsai, C.‐F. , Lin, W.‐C. , Chang, W.‐H. , Chen, T.‐J. , Pan, T. L. , Su, T. P. , & Bai, Y. M. (2014). Risk of dementia among patients with asthma: A nationwide longitudinal study. Journal of the American Medical Directors Association, 15(10), 763–767. 25037169 10.1016/j.jamda.2014.06.003

[brb370048-bib-0010] Gandhi, A. P. , Shamim, M. A. , & Padhi, B. K. (2023). Steps in undertaking meta‐analysis and addressing heterogeneity in meta‐analysis. The Evidence, 1(1), 44–59.

[brb370048-bib-0011] Goel, S. , Shabil, M. , Kaur, J. , Chauhan, A. , & Rinkoo, A. V. (2024). Safety, efficacy and health impact of electronic nicotine delivery systems (ENDS): An umbrella review protocol. BMJ Open, 14(1), e080274. 10.1136/bmjopen-2023-080274 PMC10826537 38286688

[brb370048-bib-0012] Iamthanaporn, C. , Wisitsartkul, A. , & Chuaychoo, B. (2023). Cognitive impairment according to Montreal Cognitive Assessment independently predicts the ability of chronic obstructive pulmonary disease patients to maintain proper inhaler technique. BMC Pulmonary Medicine, 23(1), 144. 37101175 10.1186/s12890-023-02448-x PMC10131352

[brb370048-bib-0013] Irani, F. , Barbone, J. M. , Beausoleil, J. , & Gerald, L. (2017). Is asthma associated with cognitive impairments? A meta‐analytic review. Journal of Clinical and Experimental Neuropsychology, 39(10), 965–978. 28325118 10.1080/13803395.2017.1288802

[brb370048-bib-0014] Joh, H. K. , Kwon, H. , Son, K. Y. , Yun, J. M. , Cho, S. H. , Han, K. , Park, J. H. , & Cho, B. (2023). Allergic diseases and risk of incident dementia and Alzheimer's disease. Annals of Neurology, 93(2), 384–397. 36093572 10.1002/ana.26506

[brb370048-bib-0015] Kim, S. Y. , Min, C. , Oh, D. J. , & Choi, H. G. (2019). Risk of neurodegenerative dementia in asthma patients: A nested case–control study using a national sample cohort. BMJ open, 9(10), e030227. 10.1136/bmjopen-2019-030227 PMC6797318 31597651

[brb370048-bib-0016] Kroll, J. L. , Steele, A. M. , Pinkham, A. E. , Choi, C. , Khan, D. A. , Patel, S. V. , Chen, J. R. , Aslan, S. , Sherwood Brown, E. , & Ritz, T. (2018). Hippocampal metabolites in asthma and their implications for cognitive function. NeuroImage: Clinical, 19, 213–221. 30035015 10.1016/j.nicl.2018.04.012 PMC6051470

[brb370048-bib-0017] Langan, D. , Higgins, J. P. , Jackson, D. , Bowden, J. , Veroniki, A. A. , Kontopantelis, E. , Viechtbauer, W. , & Simmonds, M. (2019). A comparison of heterogeneity variance estimators in simulated random‐effects meta‐analyses. Research Synthesis Methods, 10(1), 83–98. 30067315 10.1002/jrsm.1316

[brb370048-bib-0018] Lovett, R. M. , Curtis, L. M. , Persell, S. D. , Griffith, J. W. , Cobia, D. , Federman, A. , & Wolf, M. S. (2020). Cognitive impairment no dementia and associations with health literacy, self‐management skills, and functional health status. Patient Education and Counseling, 103(9), 1805–1811. 32197929 10.1016/j.pec.2020.03.013 PMC7864102

[brb370048-bib-0019] MacLeod, C. A. , Bu, F. , Rutherford, A. C. , Phillips, J. , Woods, R. , & team, C. W. R. (2021). Cognitive impairment negatively impacts allied health service uptake: Investigating the association between health and service use. SSM‐Population Health, 13, 100720. 33364299 10.1016/j.ssmph.2020.100720 PMC7750552

[brb370048-bib-0020] Nair, A. K. , Van Hulle, C. A. , Bendlin, B. B. , Zetterberg, H. , Blennow, K. , Wild, N. , Kollmorgen, G. , Suridjan, I. , Busse, W. W. , Dean, D. C. , & Rosenkranz, M. A. (2023). Impact of asthma on the brain: Evidence from diffusion MRI, CSF biomarkers and cognitive decline. Brain Communications, 5, fcad180. 37377978 10.1093/braincomms/fcad180 PMC10292933

[brb370048-bib-0021] Nair, A. K. , Van Hulle, C. A. , Bendlin, B. B. , Zetterberg, H. , Blennow, K. , Wild, N. , Kollmorgen, G. , Suridjan, I. , Busse, W. W. , & Rosenkranz, M. A. (2022). Asthma amplifies dementia risk: Evidence from CSF biomarkers and cognitive decline. Alzheimer's & Dementia: Translational Research & Clinical Interventions, 8(1), e12315. 35846157 10.1002/trc2.12315 PMC9270636

[brb370048-bib-0022] Peng, Y.‐H. , Wu, B.‐R. , Su, C.‐H. , Liao, W.‐C. , Muo, C.‐H. , Hsia, T.‐C. , & Kao, C.‐H. (2014). Adult asthma increases dementia risk: A nationwide cohort study. Journal of Epidemiology and Community Health, 69, 123–128. 25271249 10.1136/jech-2014-204445

[brb370048-bib-0023] Ren, M. , Feng, M. , Long, Z. , Ma, J. , Peng, X. , & He, G. (2021). Allergic asthma‐induced cognitive impairment is alleviated by dexamethasone. Frontiers in Pharmacology, 12, 680815. 34248632 10.3389/fphar.2021.680815 PMC8261293

[brb370048-bib-0024] Rhyou, H.‐I. , & Nam, Y.‐H. (2021). Association between cognitive function and asthma in adults. Annals of Allergy, Asthma & Immunology, 126(1), 69–74. 10.1016/j.anai.2020.08.022 32858237

[brb370048-bib-0025] Rusanen, M. , Ngandu, T. , Laatikainen, T. , Tuomilehto, J. , Soininen, H. , & Kivipelto, M. (2013). Chronic obstructive pulmonary disease and asthma and the risk of mild cognitive impairment and dementia: A population based CAIDE study. Current Alzheimer Research, 10(5), 549–555. 23566344 10.2174/1567205011310050011

[brb370048-bib-0026] Shamim, M. A. , Gandhi, A. P. , Dwivedi, P. , & Padhi, B. K. (2023). How to perform meta‐analysis in R: A simple yet comprehensive guide. The Evidence, 1(1), 60–80.

[brb370048-bib-0027] Wang, T. , Huang, X. , & Wang, J. (2023). Asthma's effect on brain connectivity and cognitive decline. Frontiers in Neurology, 13, 1065942. 36818725 10.3389/fneur.2022.1065942 PMC9936195

[brb370048-bib-0028] Wang, Y. , Mou, Y.‐K. , Wang, H.‐R. , Song, X.‐Y. , Wei, S.‐Z. , Ren, C. , & Song, X.‐C. (2023). Brain response in asthma: The role of “lung‐brain” axis mediated by neuroimmune crosstalk. Frontiers in Immunology, 14, 1240248. 37691955 10.3389/fimmu.2023.1240248 PMC10484342

